# Low-Cost Sensor for Continuous Measurement of Brix in Liquids

**DOI:** 10.3390/s22239169

**Published:** 2022-11-25

**Authors:** Swapna A. Jaywant, Harshpreet Singh, Khalid Mahmood Arif

**Affiliations:** 1 Department of Mechanical and Electrical Engineering, SF&AT, Massey University, Auckland 0632, New Zealand; 2 New Zealand Product Accelerator, The University of Auckland, Auckland 1010, New Zealand

**Keywords:** Brix sensor, pressure sensor, hydrometer, differential pressure, specific gravity, wine fermentation

## Abstract

This paper presents a Brix sensor based on the differential pressure measurement principle. Two piezoresistive silicon pressure sensors were applied to measure the specific gravity of the liquid, which was used to calculate the Brix level. The pressure sensors were mounted inside custom-built water-tight housings connected together by fixed length metallic tubes containing the power and signal cables. Two designs of the sensor were prepared; one for the basic laboratory testing and validation of the proposed system and the other for a fermentation experiment. For lab tests, a sugar solution with different Brix levels was used and readings from the proposed sensor were compared with a commercially available hydrometer called Tilt. During the fermentation experiments, fermentation was carried out in a 1000 L tank over 7 days and data was recorded and analysed. In the lab experiments, a good linear relationship between the sugar content and the corresponding Brix levels was observed. In the fermentation experiment, the sensor performed as expected but some problems such as residue build up were encountered. Overall, the proposed sensing solution carries a great potential for continuous monitoring of the Brix level in liquids. Due to the usage of low-cost pressure sensors and the interface electronics, the cost of the system is considered suitable for large scale deployment at wineries or juice processing industries.

## 1. Introduction

In many industries, such as distillery, juice processing, maple and corn syrup production, the outcome always relies on a solution with sugar (sucrose, glucose, fructose, etc.) as the major solute. Similarly, in brewing and wine making, the final fermented product is dependent on the starting sugar content of the must and the way it changes during fermentation. Therefore, it is essential to monitor the sugar content in these industries, preferably in a continuous manner [1]. Dissolved sugar in beverages is indicated in terms of Brix, Baume, or Oechsle. However, Brix is the commonly used unit in the process industry. It indicates the number of dissolved solids in a liquid measured via its specific gravity (SG) [2]. One degree Brix is 1 g of sucrose in 100 g of solution (1 °Bx = 1% sugar) [3]. Brix can be measured using sample-based measurement or in-line measurement. There is a linear relationship between SG and density, hence Brix measurement can be done through one of these two quantities [4]. SG or density can be measured using various methods, such as hydrometer principle [5], optical methods based on refractive index [1] or infrared absorbance, near-infrared spectroscopy, X-ray absorption, ultrasonic method [6], fibre Bragg grating [7], mass flow of liquid based on Coriolis principle [8], differential pressure measurement [9], or biosensing [10].

Hydrometer is the most commonly used, hand-held, and economical device to measure SG. However, it includes a manual measurement process that can cause an incorrect reading of the meniscus. In addition, temperature compensation is required in the final reading as its operation is temperature dependent [11,12,13]. These constraints are overcome by a refractometer, which works on the principle of refraction of light. Both analogue and digital versions of refractometer are available but analogue type is generally a cost-effective option. Since analogue refractometers are not very accurate due to subjective reading, digital and benchtop refractometers are the preferred versions in the industry despite being expensive compared to their analogue counterparts [4]. Electronic tongue is another modern portable and cost-effective solution that provides a quick Brix measurement; however, its low selectivity is a major issue [14,15,16]. Surface plasmon resonance (SPR)-based sensors and chromatography are also among the sample-based methods used for Brix measurement. In summary, sample-based methods are manual, time-consuming, and most importantly cannot be the solution of choice for continuous monitoring [4].

Several in-line or continuous monitoring Brix sensors have also been developed. Among them, vis/NIR spectroscopy is a widely used method, especially in wineries, as it can allow the additional benefit of composition analysis [17,18]. It is possible to monitor the concentration and evolution of phenolic compounds during wine fermentation [19]. Ultrasonic velocity measurements have also been demonstrated as a successful method for real-time measurement as density and sound velocity relate linearly [20,21]. In the ultrasonic method, automation can be easily implemented, leading to a complete analyser [22]. Measurement of liquid density via the Coriolis principle is another method for in-line real-time monitoring of liquid sugar levels. The liquid is made to pass through oscillating tubes; the mass flow rate of the liquid affects the oscillation of the tubes, and this is used to calculate the liquid density [23]. However, the applied static pressure causes inaccuracies in measuring the liquid density.

There are commercially available in-line measurement sensors, such as Liquiphant M vibrating fork, Fermetrol probe, Micro-LDS, and Tilt hydrometer [24]. Liquiphant M vibrating fork utilises the density measurement principle. In Fermetrol probe, a semipermeable water-responsive polymer is used to find out the osmotic potential caused by the concentration of sugar in the must. Micro-LDS is based on the Coriolis effect. However, these methods are expensive solutions normally only suitable for larger industries. Sensor cleaning is another problem during and after fermentation. In biosensors, enzyme stability highly affects parameters such as accuracy, reproducibility, and operational lifetime. Tilt hydrometer is an economical free-floating digital hydrometer. However, it is specifically designed for home brewing and is not suitable for large industries [4].

In this paper, we present a low-cost liquid Brix measurement sensor based on the differential pressure measurement principle. The sensor is intended for continuous measurement of Brix to monitor processes such as wine fermentation, brewing, etc. The use of differential pressure for level [9] and density measurement [25] of liquids is well known, however a systematic design and testing approach has not been demonstrated in relation to the Brix measurements. In the proposed technique, two pressure sensors are applied to measure the differential pressure, which is used to compute specific gravity and then the Brix value of the liquid. The rest of the paper describes the measurement principle, sensor design, experimental setup, and results from the two different experiments.

## 2. Methodology

### 2.1. Principle of Measurement

**Figure 1:**
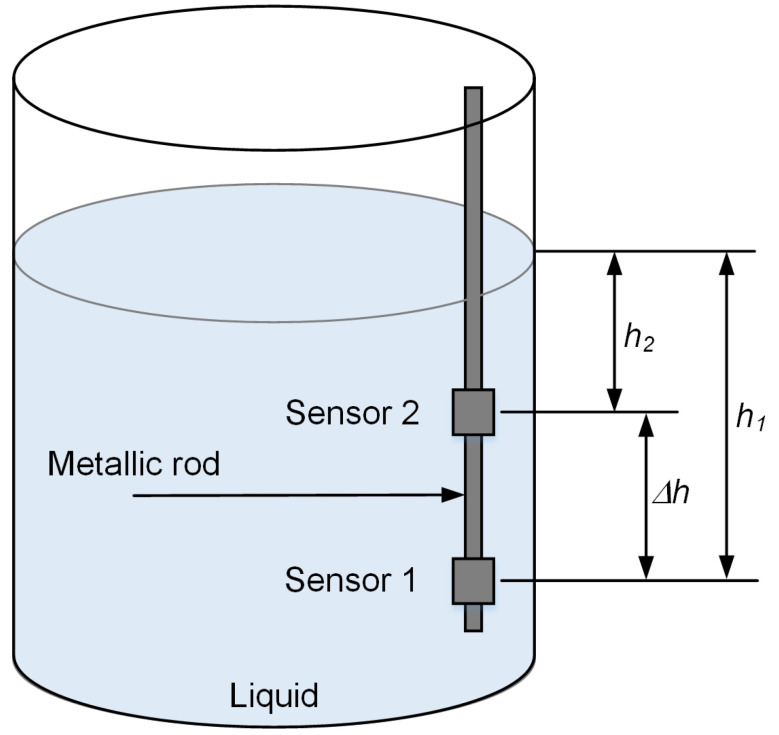
Differential pressure measurement in a liquid container.

A schematic illustration of the differential pressure measurement in a liquid tank is shown in Figure 1. The relationship between pressure and density of the liquid is given by

(1) P=ρgh,

where *P* is the pressure, ρ is the density of the liquid, *g* is the acceleration of gravity with a value of 9.8 m/s^2^, and *h* is is the level of the liquid in the container. Equation (1) indicates that the pressure at any point inside the liquid is directly proportional to the density of the liquid.

If two pressure sensors are used in the system, as shown in Figure 1, the pressures measured by the sensors can be represented by

(2) $$P_{1}=\rho gh_{1},$$

and

(3) $$P_{2}=\rho gh_{2},$$

where *P*_1_ and *P*_2_ are the pressures measured by sensor 1 and sensor 2, respectively. The depths of sensor 1 and sensor 2 are *h*_1_ and *h*_2_.

The differential pressure between two sensors can be calculated as

(4) $$\Delta P=P_{1}-P_{2}=\rho g\left(h_{1}-h_{2}\right)=\rho g\Delta h,$$

where Δ*P* denotes the differential pressure, and Δ*h* denotes the distance between two sensors. Thus, the density of the liquid can be determined by calculating the differential pressure as

(5) $$\rho =\frac{\Delta P}{g\Delta h}.$$

The density value from Equation (5) is used to calculate the specific gravity of the liquid as follows:

(6) $$SG=\frac{\rho }{\rho_{w}},$$

where SG is the specific gravity of the liquid, ρ is the density of the liquid, and ρ_w_ is the density of the water, which is 1000 kg/m^3^ at 4 °C.

Finally, the following formula is used to calculate the Brix value [26].

(7) $${}^{\circ }Bx=\left(SG-1\right)/0.004$$

### 2.2. Pressure Sensor Selection

Pressure sensor selection is the most important step when designing the proposed Brix sensor. The major criteria considered while selecting the pressure sensor are the pressure range, measurement resolution, output interface, and packaging type. The pressure range is dependent upon the depth at which the sensors are immersed in the testing tank. In the process industry, tank height can vary from 1 m up to 5 m. Hence, a pressure sensor with a range of 0 to 25 psi is desired if the sensors are immersed in the tank for the full depth. However, this is not required for Brix measurement as the sugar content is uniform in the whole tank and measurements at any depth are representative of the solution’s Brix value. This means a pressure sensor with a smaller range can be used in the proposed method of measurement.

To obtain the accuracy of ± 0.1 °Bx, the preferred resolution of the pressure sensor needs to be 0.001 psi. This is determined by calculating the pressure at a fixed depth for 0 °Bx and 0.1 °Bx. For example, at 2 m depth, the pressure is 2.8447 psi at 0 °B if the density of the liquid is 1000 g/L and the acceleration of gravity is 9.80665 ms^−2^. However, at 0.1 °Bx the density changes to 1000.39 g/L, producing a pressure of 2.8458 psi. The difference of pressures (2.8458–2.8447) is 0.0011.

The output interface and packaging type of the pressure sensor are important as they relate to how the sensors are interfaced with the measurement electronics and mounted inside the housing. Sensors with analogue or digital interfaces are available and can be used; however, an analogue sensor requires analogue to digital conversion (ADC) and other signal conditioning for interfacing with a microcontroller. The packaging type can be flush mount or port type, but flush mount is preferred as it is less susceptible to residue build up and clogging. Furthermore, the sensor should be suitable for liquid media and temperature-compensated, with an operating temperature range up to 80 °C. Table 1 lists the sensors shortlisted according to the selection criteria.

Sensor MPRLS0025PA00001A is a port type sensor with digital output. In real life application, residues/ material can be stuck in the port, which can cause fouling of the sensor, and cleaning of the sensor will become challenging. Some additional packaging may be required to avoid this problem. Hence, preference is given to the TE connectivity sensors. The output of the sensor 86-015G-C is analogue voltage, which requires additional signal conditioning circuitry (e.g., amplification and analogue to digital conversion). On the other hand, 86BSD015PG provides digital output directly in response to the applied pressure. Due to the digital signal output, flush mount, and lower cost compared to 86-015G-C, 86BSD015PG is selected.

### 2.3. Sensor Design

**Figure 2:**
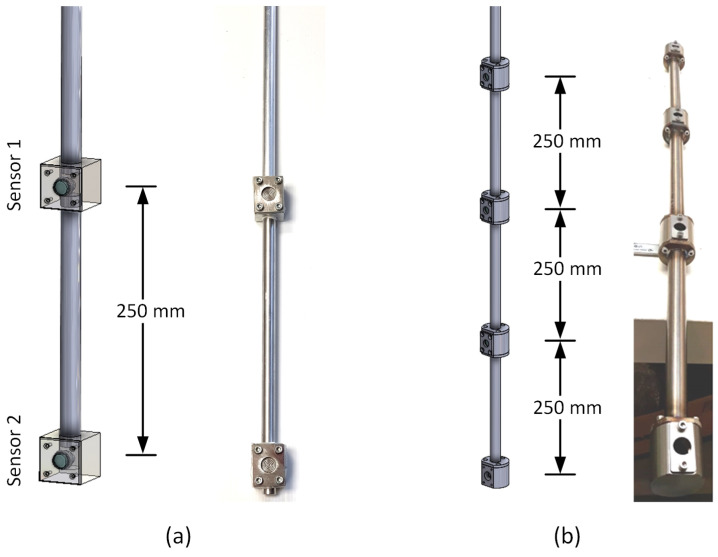
Brix sensor design: (**a**) consisting of two pressure sensors for sugar monitoring and (**b**) consisting of four pressure sensors for fermentation monitoring.

**Figure 3:**
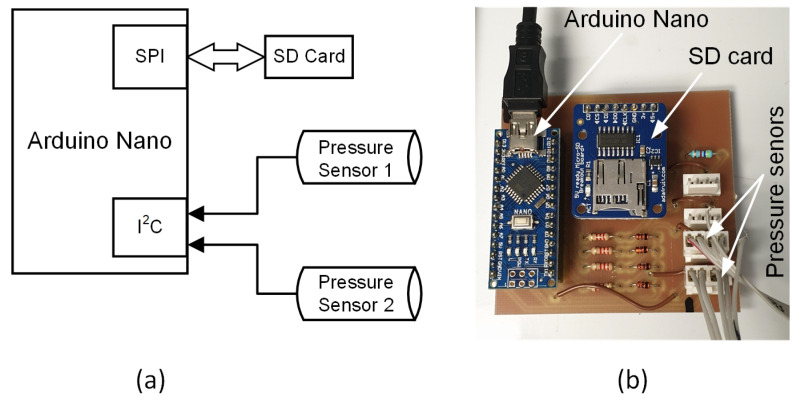
Sensor interface electronics. (**a**) Block diagram of the measurement electronics. (**b**) Actual measurement board.

Figure 2a shows the schematic of the Brix sensor used in the sugar monitoring experiments in the lab. It includes two piezoresistive silicon pressure sensors (86BSD015PG) and a custom-built sensor mount made of aluminium. The distance between the pressure sensors is kept constant at 250 mm, which produces the pressure difference, meeting the resolution requirements. The pressure sensors are placed inside the housings for the sensors, as shown. The data cable of each sensor passes through the hollow rod of the mount and is connected to the microcontroller (see Figure 3). Table 2 shows the major technical specifications of the piezoresistive silicon pressure sensor used in the Brix sensor. The pressure sensor provides a 14-bit digital output and is designed for o-ring mounting. Figure 2b illustrates the schematic of the Brix sensor used in fermentation monitoring. This sensor includes four piezoresistive silicon pressure sensors (86BSD015PG) to allow for the calculation of differential pressure at different inter sensor distances (250 mm, 500 mm, and 750 mm) in the fermentation experiment and to confirm that differential pressure is independent of the distance between the sensors. The material of the sensor mount is stainless steel, which is ideal for food grade applications.

The block diagram of the measuring circuit is shown in Figure 3a. It includes pressure sensors, an SD card reader, and an Arduino Nano microcontroller board. The pressure sensors output data on I^2^C port is directly connected to the I^2^C bus of the Arduino board. The SD card is used to store the sensor’s output and it is connected to the SPI bus of the Arduino board. Figure 3b shows the actual prototype of the board used for the measurements.

**Figure 4:**
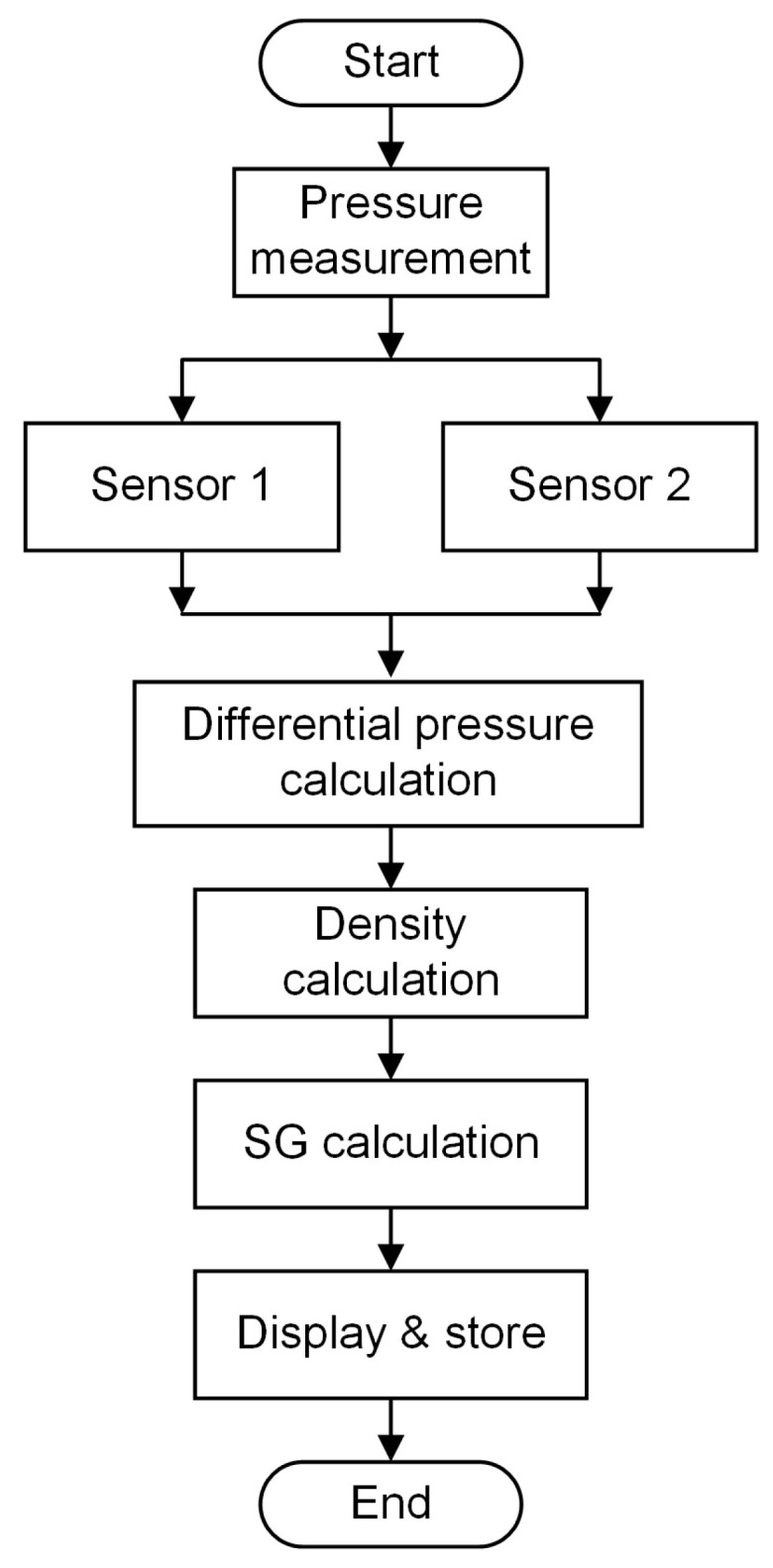
The flow chart of the Brix measurement process.

The pressure read from the sensors is processed in the microcontroller to calculate the density and Brix value of the liquid. The data is stored on the SD card in the CSV format. The flow chart in Figure 4 shows the sequence of steps involved in the whole measurement process.

### 2.4. Linearization

In general, pressure sensors measure stress over a surface area (diaphragm) that is not totally uniform over the measurement range of the sensors and leads to non-linear output. Therefore, least squares regression is selected and implemented in the embedded algorithm to improve the linearity.

To obtain the best fit of the line, the following linear polynomial is used

(8) y=ax+b,

where *y* represents the theoretical value of the sensor, *x* is the actual value of the sensor, *a* is the gradient, and *b* is an intercept of the linear polynomial.

The least squares regression minimizes the error between the each theoretical data and calibration data. Therefore, the partial derivatives of a and b are zero and are expressed as [9]

(9) $$a=\frac{N\Sigma x_{i}y_{i}-\Sigma x_{i}\Sigma y_{i}}{N\Sigma x_{i}^{2}-{\left(\Sigma x_{i}\right)}^{2}},$$

and

(10) $$b=\frac{\Sigma y_{i}-a\Sigma x_{i}}{N},$$

where

*N* is the number of points,

$\Sigma x_{i}=x_{1}+x_{2}+\ldots x_{N}$,

$\Sigma y_{i}=y_{1}+y_{2}+\ldots y_{N}$,

$\Sigma x_{i}y_{i}=x_{1}y_{1}+x_{2}y_{2}+\ldots x_{N}y_{N}$,

$\Sigma {x_{i}}^{2}={x_{1}}^{2}+{x_{2}}^{2}+\ldots {x_{N}}^{2}$.

From the sensors’ actual values, the sensors’ linear polynomials are calculated, as shown in Table 3.

## 3. Experimental Setup and Results

Initially, all six pressure sensors were calibrated against the standard pressure. The results of the calibrations are shown in Table 4. The results indicate that for each increase in applied standard pressure, there is a linear increase in the output of every sensor.

### 3.1. Sugar Monitoring

**Figure 5:**
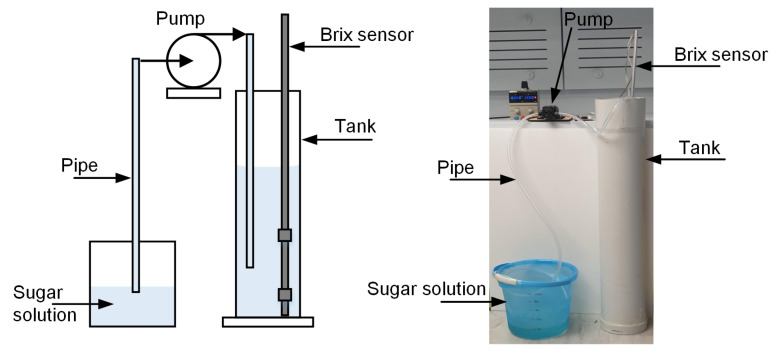
Experimental set up for sugar monitoring in the lab.

Figure 5 illustrates the Brix measurement setup with a liquid tank and the prototype of the proposed Brix sensor. A PVC pipe with a diameter of 150 mm and a length of 1 m was used as the tank. Ten different sugar solutions with Brix values ranging from 0 to 22 °Bx were prepared. Initially, a 2 °Bx sugar solution was prepared by adding 200 g of sugar to 10 L of water. A motorised hand-mixer was used for continuous stirring to uniformly dissolve the sugar into the water. The sugar solution from the container was transferred into the tank with the help of a pump. A micro-diaphragm pump (12 volts, 60 watts) was used for this purpose. Subsequently, every time, 200 g of sugar was added to the previously prepared sugar solution to obtain the sugar solution with a new Brix value and the respective reading was noted down.

The experiment was performed under the atmospheric pressure condition. The Brix sensor (Figure 2a) was immersed in the tank to obtain the readings. The differential pressure values across two pressure sensors were measured after every 60 s using the microcontroller board. Subsequently, corresponding SG (Equations (5) and (6)) and the Brix values of the sugar sample (Equation (7)) were calculated through the microcontroller firmware and stored in the SD card.

A commercially available hydrometer Tilt [27] was also used to measure the SG (and corresponding Brix) values of the sugar solution for comparison purposes.

#### Results and Discussion

**Figure 6:**
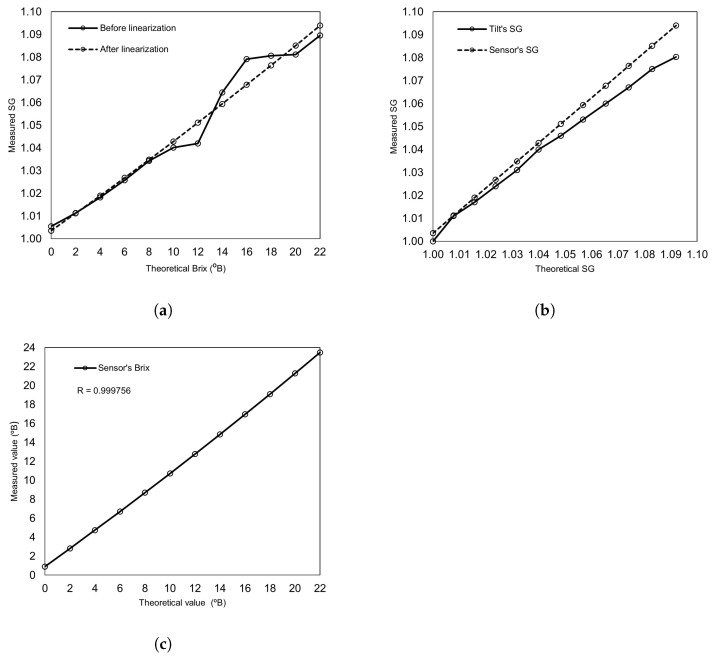
Measurements plots from the sugar monitoring experiments. (**a**) Linearization of the proposed Brix sensor; (**b**) SG values of the proposed sensor and Tilt hydrometer; (**c**) Brix measurement results (theoretical vs. measured).

Table 5 shows the result of the proposed Brix sensor and Tilt hydrometer. The output of the Brix sensor was found to be non-linear when raw data was plotted. However, linearization resulted in a linear relation, as shown in Figure 6a, between the measured SG value by the sensor for the corresponding Brix sample solution. The non-linearity of the measured values was removed using the LSG algorithm. The gradient and intercept values of the linear polynomial were calculated, and the subsequent curve-fitting equation was included in the software. The linearization caused an improvement in the accuracy of SG value from 99.56% to 99.73%.

As mentioned earlier, the SG values of the proposed sensor were evaluated with the commercially available Tilt hydrometer’s output. Figure 6b compares the proposed sensor’s SG values with the commercially available Tilt hydrometer’s SG values. The proposed sensor has a more linear response compared to the Tilt sensor. After a 1.05 SG value, Tilt’s SG value is below the actual SG value. The overall accuracy of the Tilt hydrometer SG value is 99.56%, which is less than the proposed sensor. The Tilt hydrometer gives the output in terms of the SG value. Hence, it is required to calculate the Brix value manually whereas the proposed sensor provides both the terms (SG and Brix) in the output.

Figure 6c shows the relationship between the theoretical and measured Brix values obtained from the proposed Brix sensor. The theoretical values are the expected values of Brix at different sugar concentration levels in the solution. The measured values linearly increase as the sugar level in the solution increases. However, there is some offset at the zero sugar level due to the pressure sensors not always giving zero differential output at the start. This offset is taken as a systematic value and considered insignificant.

### 3.2. Fermentation Monitoring

**Figure 7:**
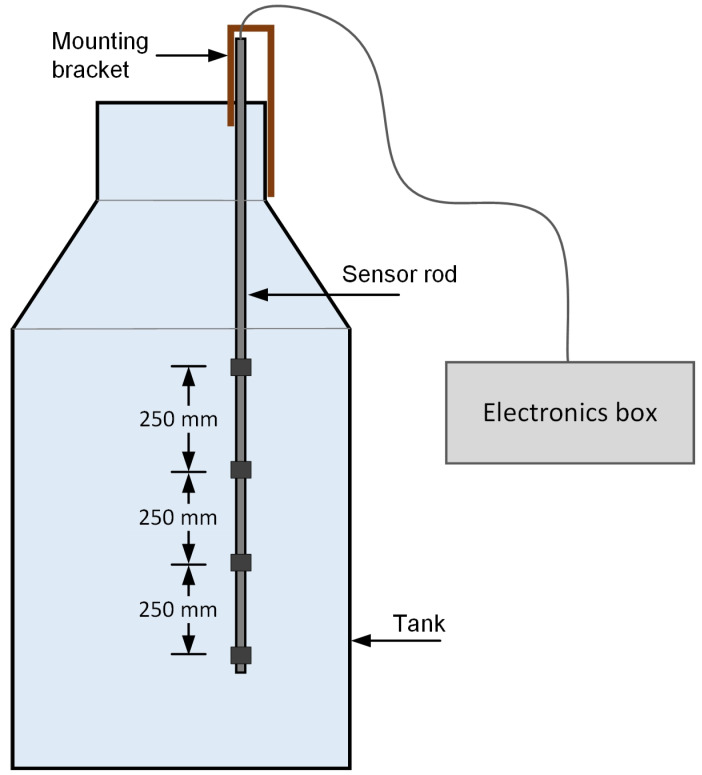
Schematic of the fermentation monitoring setup. The sensor rod is held in place by a bracket attached to the top opening of the fermentation tank.

After achieving satisfactory results in the sugar monitoring experiments, fermentation monitoring was performed to observe the sensor functionality. Fermentation was performed in a tank using a sugar solution and yeast. For this purpose, a 1000 L sugar solution was prepared by mixing 200 kg sugar into 1000 L of water. The mixture was circulated overnight with the help of a pump to dissolve sugar. The following morning specific gravity of the solution was measured with the help of a traditional hydrometer, which was 1.0848. The yeast was activated using lukewarm water and added to the sugar solution to start the process of fermentation. The proposed sensor illustrated in Figure 2b was immersed in the tank to monitor the fermentation process continuously (Figure 7).

#### Results and Discussion

**Figure 8:**
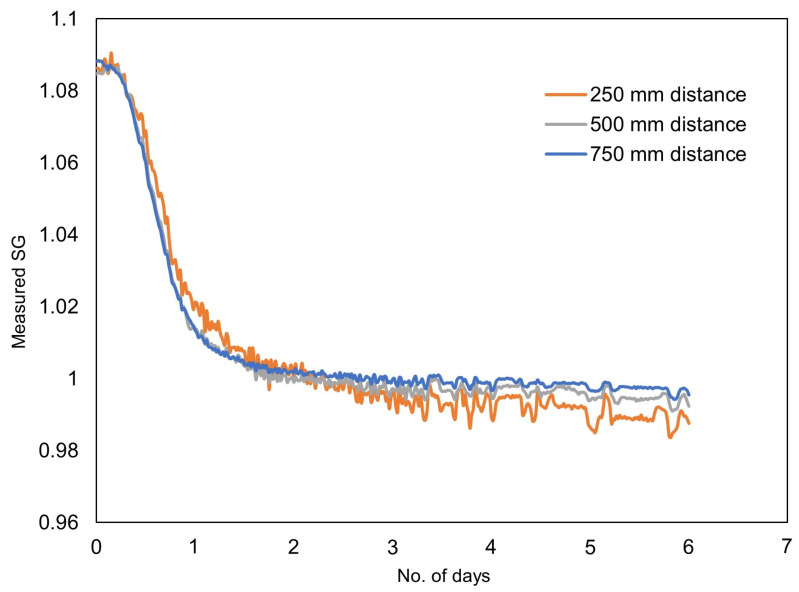
Effect of fermentation on the SG of the solution measured over 6 days with the proposed Brix sensor having pressure sensors 250 mm, 500 mm, and 750 mm apart.

**Figure 9:**
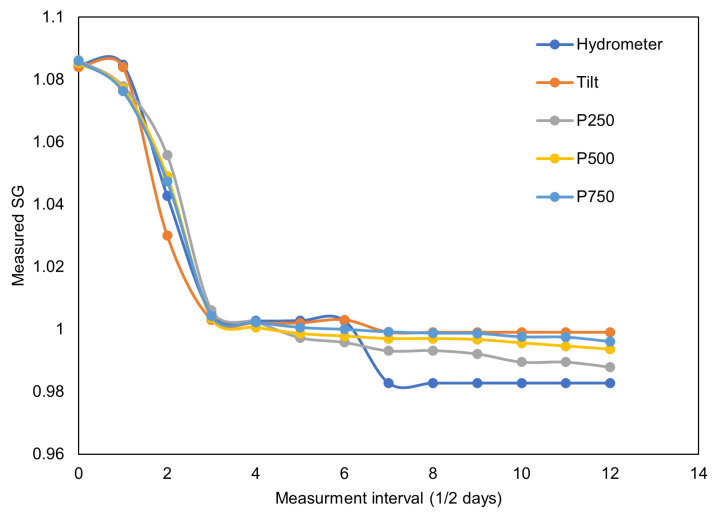
Specific gravity of the fermentation solution measured with a traditional hydrometer, Tilt hydrometer, and the proposed Brix sensor having pressure sensors 250 mm, 500 mm, and 750 mm apart.

During fermentation, the sugar present in the solution was transformed into ethanol due to the presence of yeasts. The process released carbon dioxide as a by-product. The sugar content started to decrease as fermentation progressed. The decrease in sugar level was continuously recorded in terms of the SG level by the proposed sensor. Additionally, every morning the SG of the solution was measured using a hydrometer, a Tilt hydrometer, and the proposed sensor (Table 6) for comparison purposes. During the initial two days, the sugar content decreased sharply and, after that, the process slowed down as expected. It took almost six days to drop the sugar content to a near zero level. The data recording was stopped but the fermentation continued for the seventh day as well. A total of 34,457 readings at a fixed interval of 15 s were recorded in 6 days (5760 readings per day). In the end, experimental measurements were downloaded from the SD card and represented in terms of a graph, as shown in Figure 8. A plot of the values recorded at 12 h intervals (Table 6) is shown in Figure 9.

The curves in Figure 8 match with the wine fermentation curves presented in the literature [28,29], indicating the successful application of the proposed sensor in fermentation monitoring. The measurement results from the proposed Brix sensors and the other two sensors taken at the 12 h interval also closely match, supporting the success of the proposed sensor design. Furthermore, the need for manual sampling for the traditional and Tilt hydrometers is completely eliminated in the case of the proposed sensor.

**Figure 10:**
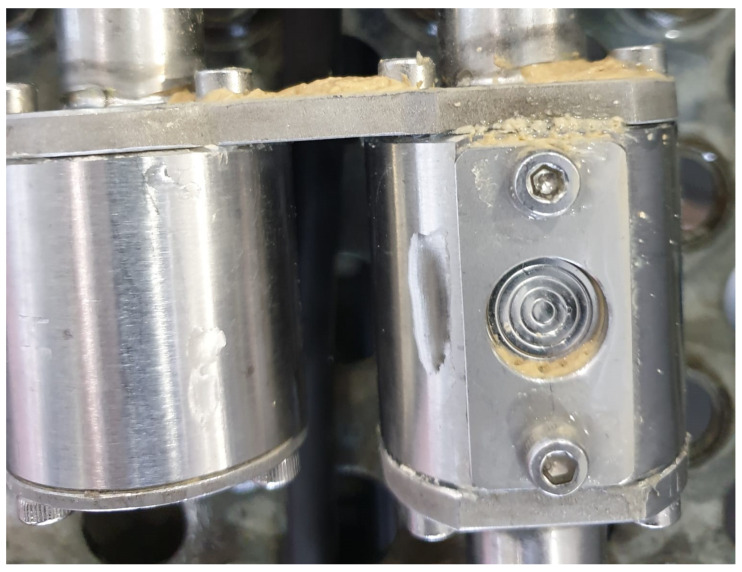
Residue build-up problem.

During fermentation monitoring, four sensors were used. Each of the sensors were connected 250 mm apart from each other. This allowed us to observe the effect of increasing the distance between two sensors on the Brix measurement. It is clear from Figure 8 and Figure 9 that the measurements are unaffected by the distance between the sensors, although there is some difference in the measurements taken at 250 mm distance. We attribute this difference to the individual noise level of the sensors. Another possible reason is the residue build up near and on the diaphragm of the pressure sensors, as shown in Figure 10.

## 4. Conclusions

Continuous in-line Brix monitoring is important in many industries, but a suitable and low-cost method does not exist. The available sample-based methods for measuring the liquid Brix value are manual, time-consuming, and most importantly cannot be the solution for continuous monitoring. On the other hand, existing in-line measurement methods are expensive or too cumbersome to use.

The Brix sensor we have presented is based on the measurement of differential pressure in the liquid. The sensor is fully automatic due to the use of an embedded microcontroller. The Brix sensor uses two pressure sensors for differential pressure measurement. Hence, the key element of the Brix sensor is the linearity and accuracy of the pressure sensors. The least-squares regression model is used to provide the linear compensation of the proposed Brix sensor. The experimental results suggest that the SG and Brix measurement in an open tank is feasible and accurate. It is independent of the liquid level in the tank. The linearization caused an improvement in the accuracy of the SG value. The results of the proposed sensor in sugar monitoring experiment are compared with a commercially available Tilt hydrometer. The proposed sensor shows the SG value more accurately than the Tilt hydrometer. The Brix value of the sensor shows some offset that exists due to the offset present in the pressure sensors.

To show the industrial application of the proposed Brix sensor, it was tested in a real fermentation experiment. The sensor successfully monitored the changing levels of Brix over a period of seven days. Multiple pressure sensors were mounted on the mounting rod to study the effect of the distance between the sensors on the resulting Brix values. It was found that the measurements were independent of the distance between the sensors used for the differential pressure calculation.This is significantly important as it allows the proposed sensor to be used as long as it is fully immersed in the liquid.

Problems such as residue build up and sensor failure due to liquid ingress were found and need to be remedied for application of the proposed sensor in actual industrial applications. Nevertheless, the sensor has proven to be a successful design and a low-cost method of monitoring Brix in liquid solutions. We assumed a homogeneous temperature in the experimental tank due to its small size. However, in future, we aim to include the temperature compensation and focus on removing the offset with the help of software algorithms. Additionally, the interface electronics will be modified to include IoT features in the sensor.

## Figures and Tables

**Table 1:** List of short-listed pressure sensors.

Sr. No.	Manufacturer	Part No.	Pressure Type	Output	Cost USD
1	TE connectivity, Berwyn, PA, USA	86BSD015PG-3AIC	0–15 psi gauge	I^2^C	74.5
2	TE connectivity, Berwyn, PA, USA	86-015G-C	0–15 psi gauge	0–100 mV	93.64
3	Honeywell, Houston, TX, USA	MPRLS0025-PA00001A	0–25 psi absolute	I^2^C	16.33

**Table 2:** Performance specifications of the piezoresistive silicon pressure sensor.

Parameter	Value	Units
Pressure range	0 to 10	PSI
Zero presput	666	Count Hex
Full scale pressure output	399A	Count Hex
Accuracy	+/− 0.25	%Span
Total error band	+/− 1	%Span
Pressure resolution	0.008	%Span
Input voltage range	3.3 (typical)	V
	2.7 (min)	V
	2.7 (max)	V
Supply current	3	mA
Load resistance	10 (min)	kΩ
Operating temperature	−40 to 125	°C
Output pressure resolution	14 (max)	Bits
Interface type	I^2^C	–

**Table 3:** Linear polynomial values of the proposed sensor.

Linear Polynomial	SSE	R^2^	RMSE
Y = 0.9830x + 0.0073	0.3855 × 10^−4^	0.969366376	0.001792504

**Table 4:** Pressure sensor calibration results.

Std Pressure	Sensor 1	Sensor 2	Sensor 3	Sensor 4	Sensor 5	Sensor 6
0	0.1	0.1	0	0	0	0
1	1.1	1.1	1	1	1	1
2	2.1	2.1	2	2	2	2
3	3.1	3.1	3	3	3	3
4	4.2	4.2	4	4	4	4
5	5.2	5.2	5.1	5	5	5
6	6.2	6.2	6.1	6.1	6.1	6.1
7	7.2	7.2	7.1	7.1	7.1	7.1
8	8.2	8.2	8.2	8.2	8.2	8.2
9	9.3	9.3	9.2	9.2	9.2	9.2
10	10.3	10.3	10.2	10.2	10.2	10.2

**Table 5:** SG and Brix values of the proposed sensor and Tilt hydrometer.

Sugar	Theoretical		Tilt Hydrometer		Proposed Sensor	
Concentration gm/mL	SG	Brix	SG	Brix	SG	Brix
0	1.0000	0	1.000	0.000	1.0035	0.8818
2	1.0078	2	1.011	2.750	1.0112	2.7985
4	1.0157	4	1.017	4.250	1.0190	4.7399
6	1.0237	6	1.024	6.000	1.0268	6.7058
8	1.0318	8	1.031	7.750	1.0348	8.6963
10	1.0400	10	1.040	10.000	1.0428	10.7114
12	1.0484	12	1.046	11.500	1.0511	12.7757
14	1.0568	14	1.053	13.250	1.0594	14.8399
16	1.0654	16	1.060	15.000	1.0678	16.9533
18	1.0741	18	1.067	16.750	1.0764	19.0912
20	1.0830	20	1.075	18.750	1.0851	21.2783
22	1.0920	22	1.080	20.083	1.0940	23.4900

**Table 6:** SG values measured by a traditional hydrometer, Tilt hydrometer, and the proposed Brix sensor.

No. of	Hydrometer	Tilt	Proposed Brix Sensor		
1/2 Days	Reading	Hydrometer	250 mm	500 mm	750 mm
0	1.0848	1.084	1.0862	1.0853	1.086
1	1.0848	1.084	1.0779	1.0772	1.0763
2	1.0428	1.030	1.0558	1.0490	1.0474
3	1.0048	1.003	1.0061	1.0035	1.0043
4	1.0028	1.002	1.0024	1.0006	1.0023
5	1.0028	1.002	0.9973	0.9986	1.0006
6	1.0028	1.003	0.9958	0.9978	1.0000
7	0.9828	0.999	0.9931	0.9970	0.9992
8	0.9828	0.999	0.9932	0.9970	0.9988
9	0.9828	0.999	0.9921	0.9967	0.9987
10	0.9828	0.999	0.9895	0.9956	0.9976
11	0.9828	0.999	0.9895	0.9946	0.9975
12	0.9828	0.999	0.9879	0.9936	0.9961

## Data Availability

Not applicable.
